# Hemorrhagic strokes in a young adult patient

**DOI:** 10.1192/j.eurpsy.2021.1947

**Published:** 2021-08-13

**Authors:** M. Suárez-Gómez, P. Rodrigues, A. Suárez Gómez

**Affiliations:** Psychiatry, ULSBA, Beja, Portugal

**Keywords:** cardiovascular risk, neurorrehabilitation, hemorragic stroke, young adult

## Abstract

**Introduction:**

Stoke is a growing public health problem in the developed world resulting in more hospitalization and mortality. In young adults stroke is the third most common cause of death world wide and the fourth leading cause of disease burden.

**Objectives:**

The aim was to describe a case of recovery after two hemorrhagic strokes in a young adult patient.

**Methods:**

It was presented a clinical case and review the current literature showing the pathway of recovery.

**Results:**

A 38-years-old man presented two episodes of hemorrahgic strokes with a lack of 6 months. With history of hypertension, smoking habits and consume of cannabinoid. The first hemorrhagic stroke had sequels of right hemiparesis. It was diagnosed with frontal arteriovenous malformation. In the second episode was submeted to frontoparietal craniotomy with total dissection of the arteriovenous malformation. After surgery he had convulsive crises that remited with valproic and levetirazetan. It did intensive rehabilitation and two months later he recovered totally. In this momente he is functional for daily lactivities, maintained the same treatment and cognitive stimulation.

**Conclusions:**

It is necessary to accomplish for healthy habits in order to prevent strokes in young people. A better prognoses may be related to a urgent and prolonged intervention and reabilitation.

**Disclosure:**

No significant relationships.

